# Effect of acrylic splints on bite force among dentulous patients

**DOI:** 10.6026/973206300210748

**Published:** 2025-04-30

**Authors:** Madhu Ranjan, Surender Kumar, Daya Shankar, Ananya Singh, Tushar Sinha, Souvir Mohan Pandey, Amit Mahuli

**Affiliations:** 1 Department of Prosthodontics and Crown & Bridge, Dental Institute, RIMS, Ranchi, Jharkhand, India; 2 Department of Prosthodontics, Hazaribag College of Dental Sciences and Hospital, Demotand, Hazaribag, Jharkhand, India; 3 Department of Public Health Dentistry and Preventive Dentistry, Dental Institute, RIMS, Ranchi, Jharkhand 834009, India

**Keywords:** Bite force, splint, maximum voluntary bite force (MVBF), patient preference

## Abstract

Splints are commonly used in dentistry while measuring the bite force for the protection of teeth. Therefore, it is of interest to evaluate the influence of using an acrylic resin splint during bite force measurement on maximum voluntary bite force (MVBF). MVBF was recorded at the right 1st molar region on 20 participants showing a significant reduction with the splint (mean difference: 21.9 N, p=0.01). Without a splint, the mean bite force was 243.25 N (SD=59.96), whereas with a splint, it dropped to 221.3 N (SD=45.76). Discomfort was the primary reason for the decrease in MVBF, with 80% of participants preferring without splint method, highlighting challenges in their application.

## Background:

Bite force refers to the force applied by the jaws when the teeth are brought together during biting and chewing. It demonstrates the effectiveness of the masticatory muscles, dental structures and the harmonious functioning of the jaws [1]. Precise evaluation of the maximum voluntary bite force (MVBF) is essential in various dental fields, including the evaluation of prosthetic devices, diagnosis of temporomandibular disorders (TMD) and understanding the mechanics of mastication [2, 3-4]. Recent advancements in bite force measurement techniques have highlighted the importance of recording devices and methodologies in obtaining reliable data [5]. Understanding a patient's biting force in dentistry can assist doctors in identifying and treating a range of masticatory system disorders, including malocclusion, temporomandibular joint dysfunction and mandibular fractures and to evaluate the therapeutic impact of various dental procedures, including orthodontics, periodontics and tooth restorations [6, 7-8]. Also, the analysis of bite force assists in the modification of the biting force distribution and occlusion to produce a stable, functional occlusion that reduces the possibility of traumatic occlusion and occlusal interferences [9]. Thus, the knowledge of bite force is imperative in dentistry. In the vast body of literature pertaining to dentistry and oral health, there exists a broad spectrum of documented values for biting force [10]. This wide range is not arbitrary, but rather, it is influenced significantly by a combination of physiological and methodological factors, each contributing to the observed diversity [11]. On one hand, the methodological factors stem from the variety of techniques and tools employed to record biting force. These range from simple, mechanical devices such as springs, to more advanced, technologically sophisticated electronic gadgets. The choice of technique and tool can greatly impact the measured value of biting force, hence contributing to the range observed in the literature [10, 11, 12-13]. On the other hand, the physiological factors are inherent to the individuals participating in these studies. These include general physiological and anatomical characteristics such as craniofacial morphology, which is the shape and structure of the individual's skull and face. Other factors include the age and gender of the participant, both of which can influence muscle strength and thus biting force. The periodontal support of the teeth, which refers to the health and integrity of the gums and other structures supporting the teeth, also plays a role. Lastly, the overall dental state of the participant, including the presence of any oral health issues or conditions, must be taken into account [11, 12, 13, 14, 15].

Among the various methodologies employed in bite force measurement, the use of acrylic resin splints has emerged as a common practice to stabilize the measurement setup and ensure consistent results [11]. Acrylic resin splints are custom-made devices that fit over the occlusal surfaces of teeth, providing a uniform contact area and distributing occlusal forces evenly [16]. However, the influence of these splints on MVBF values remains a subject of debate, with some studies suggesting that they may alter the natural bite force patterns and others indicating no significant impact [17, 18]. Several studies have investigated using acrylic resin splints in bite force measurement. Manly and Braley were among the first to explore splints to stabilize the jaw during bite force measurement, finding that splints provided more consistent results than direct measurements [19]. More recent research by Cosme *et al.* has supported these findings, suggesting that bruxism does not significantly influence maximal bite force in young dentate adults [20]. However, some researchers argue that using splints may alter the natural occlusal patterns and lead to overestimation or underestimation of MVBF [21, 22-23]. Therefore, it is of interest to evaluate the influence of using an acrylic resin splint during bite force measurement on maximum voluntary bite force.

## Materials and Methods:

## Study design:

This study was designed as a cross-sectional observational study and has received ethical clearance from the institutional ethics committee (RIMS IEC letter number 391, dated 02.12.2024). The study was conducted from May 2024 to July 2024. Participants were selected using a convenience sampling method from the population of patients visiting the institute.

## Sample size estimation:

The sample size was calculated based on a previous study with 80% power, α= 0.05 and 95% confidence interval [24]. The sample size estimated was 20 participants. Informed consent was obtained from all participants in accordance with the Helsinki Declaration of 1975. Inclusion criteria were participants of both genders in the age group of 18 to 40 years who were physically and mentally fit, having a complete set of teeth in both the arches (the existence of a third molar, or wisdom tooth, was not taken into account) and gave consent to be included as participants.

The exclusion criteria were the following: 

[1] Any issues such as missing teeth, dental caries, restorations, heavily damaged crowns, non-vital teeth, trauma, enamel cracks, developmental defects, or congenital anomalies.

[2] Experiencing pain or tenderness around the affected teeth, TMJ, jaw and surrounding muscles.

[3] Having fillings, root canal treatments, or any dental prostheses.

[4] Recent orthodontic treatments, orthognathic surgeries, or jaw fracture treatments.

[5] Any ongoing dental treatments that could be impacted.

[6] Individuals with abnormal occlusion.

## Methodology:

After collecting the demographic data, alginate impressions (Zelgan by Dentsply Sirona, India) of both arches were made and poured with type III dental stone (Neelkanth India). The casts were mounted on a semi-adjustable articulator after face bow transfer. A splint approximately 2 mm thick was fabricated on the right side of the upper and lower arch, covering the occlusal surfaces of all posterior teeth using self-cure acrylic resin (DPI India). The occlusal surfaces of the splints were made flat and had indentations of the biting portion of the measuring device. The acrylic splints were finished, polished and positioned over the casts until the measurements of MVBF. Maximum voluntary bite force was assessed using a handheld measurement device called "BYTE" (Indian patent number 489519) [13, 25 and 26]. Participants were seated comfortably in the dental chair, sitting upright with supported backs and no head support, ensuring the Frankfurt horizontal plane was parallel to the floor and their feet resting flat [10, 11, 13 and 24]. They were trained to achieve their highest bite force prior to the actual measurement. After thorough training and comprehension, the final MVBF was recorded. Two sets of readings were taken for each patient. First without a splint on right 1st molar and second with a splint on the same side. The device's head portion was covered with a disposable sleeve. The biting part of the device was aligned over the right first molar and patients were directed to exert their maximum biting force for 3-4 seconds, ensuring they kept their heads still. A total of three readings were taken. A gap of 2-3 minutes was given between each reading to allow the musculature to relax. The highest value recorded for that specific participant was noted. The second set of readings was taken after 30 min. The acrylic splint was placed and the procedure for bite force measurement described earlier was repeated. The highest value recorded for that participant was noted. After recording the MVBF by both methods, participants were asked which method they preferred in terms of comfort, with a splint or without a splint. They were also asked the reason for their preference. The data were tabulated and statistically analyzed.

## Statistical analysis:

Descriptive statistical analysis was conducted utilizing IBM SPSS software, Version 20.0 (IBM Corp., Armonk, NY, USA). This was followed by a paired sample t-test for further analysis.

## Results:

A total of 20 patients meeting inclusion criteria were recruited in the study with equal male and female distribution. Table 1 shows the distribution of age and bite force with and without splint, Table 2 shows patients' preference for recording methods and Table 3 shows the frequency for reasons of the preference of the recording method. A total of 20 patients participated in the study, with an equal distribution of males and females. The participants' ages spanned from 20 to 40 years, with an average age of 30.8 years. (SD = 6.04). Bite force was measured with and without the splint. Without a splint, the mean bite force was 243.25 N (SD = 56.96), with values ranging from 110 N to 326 N. With splint, the mean bite force was 221.3 N (SD = 45.76), with values ranging from 106 N to 289 N. A paired sample t-test revealed a statistically significant difference in bite forces between the conditions with and without the splint (t = 2.87, p = 0.01), with the mean difference being 21.9 N (95% CI: 5.97 to 37.9) (Table 4 and Table 5). Eighty percent of participants (16 out of 20) preferred the method without a splint, while 20% (4 participants) preferred the method with the splint.

The reasons for the participants' preferences were categorized as follows:

[1] Comfortable without splint while biting: 35% (7 participants).

[2] Comfortable without splint while biting also experienced pain with splint: 30% (6 participants).

[3] Comfortable without splint while biting but experienced pain in the TMJ with splint: 15% (3 participants).

[4] Comfortable with splint while biting: 10% (2 participants).

[5] Comfortable with splint while biting but experienced pain without splint: 10% (2 participants).

The bite force was notably greater without the splint compared to with the splint. Most participants (80%) found the recording method without the splint to be more comfortable, citing pain or discomfort as the primary reason for avoiding the splint. The paired t-test confirmed a notable difference in bite forces, reinforcing the impact of the splint on bite force reduction. This analysis provides a clear comparison of bite force and participant preferences, emphasizing the challenges associated with splint usage in recording bite force.

## Discussion:

This study aimed to assess the effect of a splint on bite force in a group of 20 participants, equally divided by gender and aged between 20 and 40 years. The device utilized for measuring bite force in this research is designated as "BYTE". This portable, handheld and lightweight apparatus has been employed in prior studies, demonstrating consistent results. It comprises a head section that integrates a piezoresistive sensor and a body featuring an LCD screen that displays the measured bite force values. The biting component is circular, with dimensions of 12 mm in height and 10 mm in diameter. Overall, the device is user-friendly and has exhibited reliability in previous research, making it an optimal choice for our study [13, 25 and 26]. The findings reveal a significant reduction in bite force when using a splint compared to not using one. The mean bite force without the splint was 243.25 N, whereas with the splint, it decreased to 221.3 N, a statistically significant reduction of 21.9 N (t = 2.87, p = 0.01). The participants' preferences support these findings, with 80% favouring the method without the splint. The reasons given include comfort and the absence of pain: 35% found it more comfortable to bite without a splint and 30% experienced pain in and around the teeth with the use of a splint during the application of MVBF. Additionally, 15% reported pain in the temporomandibular joint (TMJ) when using the splint. A minority (20%) preferred using the splint, with 10% finding it more comfortable and another 10% experiencing pain in and around the teeth without it. These results highlight the subjective nature of comfort and pain perception among individuals. 

Comparatively, previous studies have shown similar trends. For instance, Gholampour *et al.* (2019) found that occlusal splints markedly decreased stress and deformation in the jawbone of bruxism patients also noted a reduction in bite force [27]. Similarly, Seiler *et al.* (2024) reported that different splint designs affected muscle activation and temporomandibular joint space variation during clenching, with some designs leading to reduced bite force [28]. Overall, the study demonstrates a higher bite force without a splint, with most participants finding this method more comfortable. The reduction in bite force with a splint suggests a trade-off between stabilization and force generation, which has clinical implications. These results are contradictory to the results shown in the research done by Waltimo, Könönen and Kleinfelder and Ludwig who stated that utilizing an acrylic splint might enhance bite force values [21, 24]. Also as concluded by Waltimo *et al.* the larger the periodontal ligament area greater will be the bite force [24]. The preference for recording bite force without a splint underscores potential issues with splint comfort and pain, which may affect patient compliance and the accuracy of measurements.

## Limitation:

To address the limitations of the current study, it is imperative to conduct further research with a substantially larger sample size to enhance the generalizability and robustness of the findings. Moreover, the implementation of a randomized controlled trial is essential to establish causality and mitigate potential biases. Additionally, a comprehensive evaluation of the splint type, encompassing its design and material properties, is warranted to ascertain its efficacy and optimize its clinical application.

## Conclusion:

The use of an acrylic resin splint significantly reduced the maximum voluntary bite force, with the average force decreasing from 243.25 N without the splint to 221.3 N with it. Most participants attributed this reduction to discomfort caused by the splint, highlighting the challenges of using splints for bite force measurements. Hence, future research should focus on improving splint design and materials to enhance comfort and measurement reliability.

## Clinical significance:

Recognizing the importance of bite force measurement in dentistry, we can effectively measure the bite force for routine dentistry without using the splint.

## Financial support and sponsorship:

Nil.

## Figures and Tables

**Table 1:** Descriptive statistics for age, bite force with and without splint

	**Number**	**Minimum**	**Maximum**	**Mean**	**Standard Deviation**
Age	20	20	40	30.8	6.04
Bite force measured without split	20	110	326	243.25	56.961
Bite force measured with split	20	106	289	221.3	45.76

**Table 2:** Frequencies of preference regarding the recording method

**Preference regarding the recording method**	**Counts**	**% of Total**	**Cumulative %**
With splint	4	20.00%	20.00%
Without splint	16	80.00%	100.00%

**Table 3:** Frequencies of reason for the preference of the recording method

**Reason for the preference**	**Counts**	**% of Total**	**Cumulative %**
Felt comfortable with splint while biting	2	10.00%	10.00%
Felt comfortable with splint while biting, Felt pain in and around tooth/teeth without splint while biting	2	10.00%	20.00%
Felt comfortable without splint while biting	7	35.00%	55.00%
Felt comfortable without splint while biting, Felt pain in and around tooth/teeth with splint while biting	6	30.00%	85.00%
Felt comfortable without splint while biting, Felt pain in and around TMJ with splint while biting	3	15.00%	100.00%

**Table 4:** Paired sample T-test to check the mean difference in all 20 study subjects with and without splint.

								**95% Confidence Interval**	
			**statistic**	**df**	**p**	**Mean difference**	**SE difference**	**Lower**	**Upper**
Bite force measured without splint	Bite force measured with splint	Student's t	2.87	19	0.01	21.9	7.64	5.97	37.9
Note. H_a_ µ Measure 1 - Measure 2 ≠ 0									

**Table 5:** Descriptive of paired sample t-test

	**N**	**Mean**	**Median**	**SD**	**SE**
Bite force measured without splint	20	243	241	57	12.7
Bite force measured with splint	20	221	214	45.8	10.2
